# Single-crystalline boron-doped diamond superconducting quantum interference devices with regrowth-induced step edge structure

**DOI:** 10.1038/s41598-019-51596-w

**Published:** 2019-10-23

**Authors:** Taisuke Kageura, Masakuni Hideko, Ikuto Tsuyuzaki, Aoi Morishita, Akihiro Kawano, Yosuke Sasama, Takahide Yamaguchi, Yoshihiko Takano, Minoru Tachiki, Shuuichi Ooi, Kazuto Hirata, Shunichi Arisawa, Hiroshi Kawarada

**Affiliations:** 1 Faculty of Science & Engineering, Waseda University, 3-4-1, Okubo, Shinjuku-ku, Tokyo, 169-8555 Japan; 2 National Institute for Materials Science, 1-2-1, Sengen, Tsukuba, Ibaraki 305-0047 Japan; 3 The Kagami Memorial Laboratory for Materials Science and Technology, Waseda University, 2-8-26, Nishiwaseda, Shinjuku-ku, Tokyo 169-0051 Japan

**Keywords:** Electrical and electronic engineering, Electronic devices

## Abstract

Superconducting quantum interference devices (SQUIDs) are currently used as magnetic flux detectors with ultra-high sensitivity for various applications such as medical diagnostics and magnetic material microstructure analysis. Single-crystalline superconducting boron-doped diamond is an excellent candidate for fabricating high-performance SQUIDs because of its robustness and high transition temperature, critical current density, and critical field. Here, we propose a fabrication process for a single-crystalline boron-doped diamond Josephson junction with regrowth-induced step edge structure and demonstrate the first operation of a single-crystalline boron-doped diamond SQUID above 2 K. We demonstrate that the step angle is a significant parameter for forming the Josephson junction and that the step angle can be controlled by adjusting the microwave plasma-enhanced chemical vapour deposition conditions of the regrowth layer. The fabricated junction exhibits superconductor–weak superconductor–superconductor-type behaviour without hysteresis and a high critical current density of 5800 A/cm^2^.

## Introduction

Superconducting quantum interference devices (SQUIDs) are magnetic flux detectors with ultra-high sensitivity^1,2^. These devices are applied for various applications such as medical diagnostics^3–5^ and magnetic material microstructure analysis^6–8^. Current high-performance SQUIDs are generally constructed from low-temperature superconductors such as Nb/AlO_X_/Nb^9^ or Al/AlO_X_/Al Josephson junctions because of their reliability and stability. However, with the diversification of detection targets and environments, improvement of the performance of SQUIDs and the materials used in their fabrication is needed. SQUIDs fabricated using new materials and forming methods have recently attracted considerable attention. For instance, carbon nanotube SQUIDs^10^ can detect the spin state of a magnetic molecule and scanning nanoscale SQUIDs^11,12^ offer dramatically improved spatial resolution. Group-IV-doped semiconductors are also considered promising materials for high-performance Josephson devices and bottom-up junction devices made from silicon or germanium^13^. Boron-doped diamond also shows potential for fabricating high-performance superconducting devices such as SQUIDs and mechanical resonators^14^ because of its excellent characteristics and processability described below. Diamond itself is an insulator; however, boron-doped diamond exhibits p-type conductivity^15^ and also exhibits superconductivity for doped boron concentrations greater than 3 × 10^20^ cm^−3^ ^16–19^. Namely, the conductivity of diamond can be controlled from the insulating to superconducting state simply by changing the boron concentration. One of the unique characteristics of superconducting diamond is that the superconducting transition temperature (*T*_*C*_) can be controlled by changing the boron concentration and plane orientation^20,21^ the highest reported *T*_*C*_ is 10 K for a (111) single-crystalline film^22,23^. The upper critical magnetic field has also been reported to be above 10 T^22,23^. These values are comparable to those of Nb–Ti, which is widely used for superconducting device applications. Boron-doped diamond films can be epitaxially grown on single-crystalline and polycrystalline insulating diamond substrates. Thus, the fabrication of a superconducting circuit on an insulating substrate with good adhesion between the substrate and superconductor can be achieved. Moreover, growth of superconducting nanocrystalline diamond^24^ on silicon nitride has recently been reported^25^, which opens a path towards the development of new diamond device applications. In addition to the above excellent characteristics, diamond exhibits strong tolerance to oxidation, heating, and physical scratches, and its microfabrication using oxygen plasma etching or selective epitaxial growth is easy^23,26^. These characteristics suggest the suitability of superconducting diamond for various device applications such as scanning SQUID microscope and strong coupled hybrid quantum systems^27–29^ composed of nitrogen-vacancy centres^30^ and superconducting circuits.

The operating temperature of a SQUID is significant for the construction of a complex SQUID system with a simple cooling system. However, the current operating temperature of a diamond SQUID is below 2 K. The first and only diamond SQUID was reported using nanocrystalline boron-doped diamond with a bridge-structured weak-link Josephson junction^31^. The SQUID could be operated in magnetic fields as large as 4 T independent of the field direction and exhibited good sensitivity of 4 × 10^−5^ Φ_0_/(Hz)^1/2^, thus demonstrating the great potential of diamond SQUIDs. However, the operating temperature was below 2 K because of the low *T*_C_ of nanocrystalline diamond. For operation at higher temperature and higher magnetic fields, it is desirable to use single-crystalline diamond. Thus, the main purpose of this study was to develop a process for fabricating a single-crystalline diamond SQUID for operation above 2 K, an operation temperature that can be easily achieved using ^4^He gas. The previously reported method for fabricating nanocrystalline diamond cannot be applied to a single-crystalline diamond film because the nanocrystalline junction uses a grain boundary on the bridge region. Hence, we needed to find a new structure. Many Josephson junction formation techniques in multiple materials have been proposed; to construct a high-performance SQUID, a superconductor–normal conductor–superconductor (S–N–S)-type Josephson junction was desired instead of a superconductor–insulator–superconductor (S–I–S)-type junction. The critical current (*I*_*C*_) of an S–N–S-type junction is higher than that of an S–I–S-type junction^32^ and the small junction capacitance of an S–N–S junction results in non-hysteretic current–voltage (*I*–*V*) characteristics. Hence, an external shunt resistor is not required, enabling the downscaling of the SQUID to the nanoscale. In previous research, our group reported a vertical S–N–S stack structure fabricated from a single-crystalline boron-doped diamond layer^33,34^. We reported clear Shapiro steps and an *I*–*V* curve without hysteresis. However, the issues of the low critical current density (160 A/cm^2^ at 2 K) and the complex stack structure, which were unsuitable for SQUID formation, remained. In this work, we fabricated a single-crystalline boron-doped diamond Josephson junction with a regrowth step-edge structure. We focused on the fact that the *T*_*C*_ of superconducting diamond differs depending on the surface orientation and that the angle and orientation of the step structure can be controlled by adjusting the epitaxial growth conditions. This structure was intended to form a weak superconducting layer in the step region. The fabricated junction exhibited non-hysteretic superconductor–weak superconductor–superconductor (S–S′–S) behaviour, and we revealed that the origin of the weak superconductor was the formation of a (001) plane in the step region. We demonstrated the first direct-current SQUID (dc-SQUID) operation using single-crystalline diamond. The details of the fabrication process and the properties are provided in the following sections.

## Results and Discussion

### Fabrication of step-edge structure for Josephson junctions and SQUIDs

**Figure 1:**
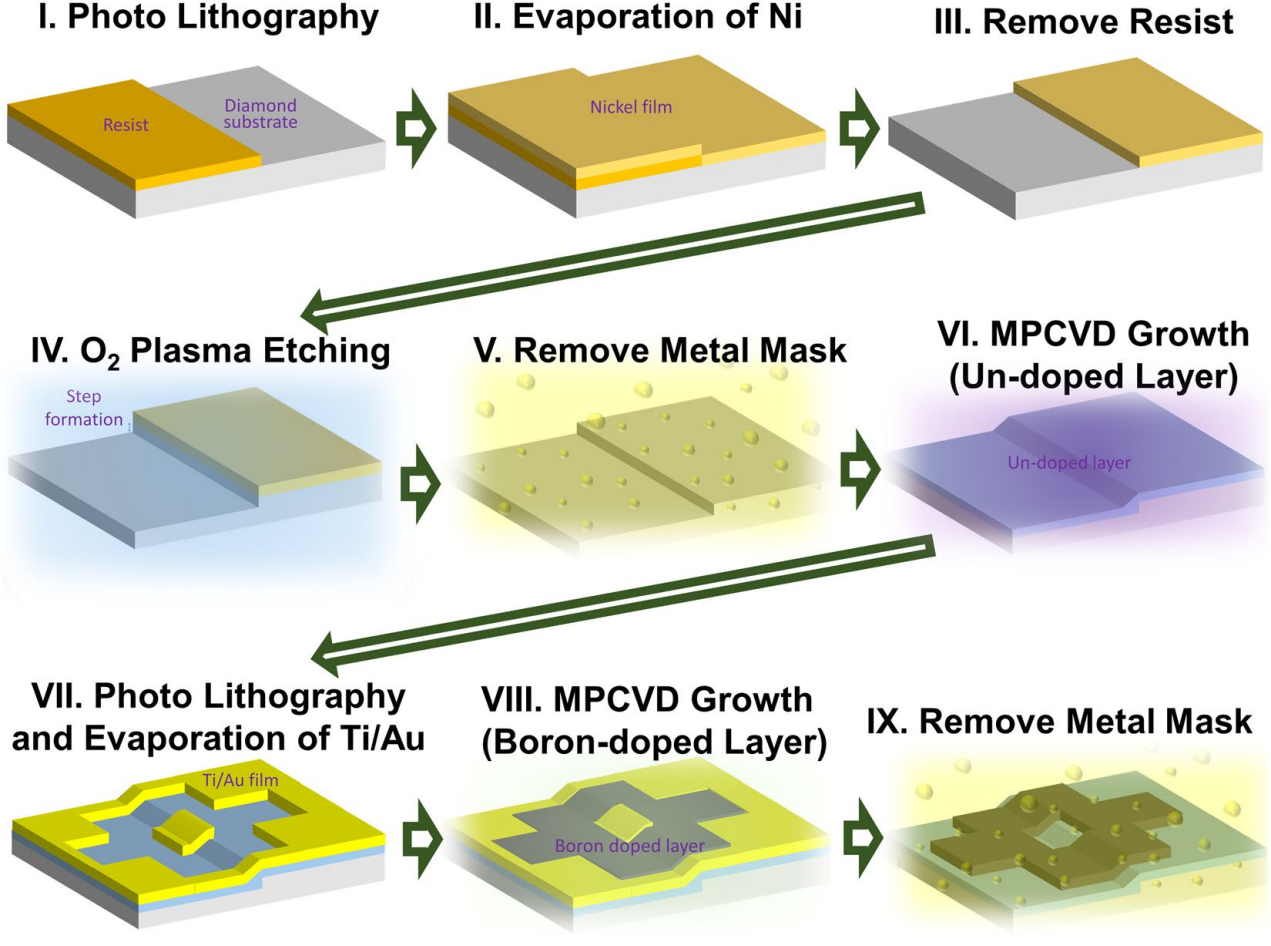
Fabrication process of single-crystalline boron-doped diamond Josephson junction and SQUID with regrowth-induced step edge structure.

The fabrication processes used to prepare the single-crystalline superconducting boron-doped diamond Josephson junctions and SQUIDs with a regrowth-induced step edge structure are illustrated in Fig. 1. First, a selective nickel metal mask was formed on a (111)-oriented single-crystalline diamond substrate using photolithography (process I), electron beam (EB) evaporation (process II), and successive lift-off (process III). A vertical step with a height (h) of 230–400 nm was formed using oxygen plasma etching (process IV) with successive removal of the metal mask by acid treatment (process V). Then, an undoped regrowth layer of 0–300 nm was homoepitaxially grown using microwave plasma enhanced chemical vapour deposition (MPCVD) (process VI). A titanium and gold Ti/Au (30/100 nm thickness) stacked metal mask pattern for the Josephson junction and SQUID was formed using photolithography, EB evaporation, and successive lift-off (process VII). A superconducting boron-doped diamond layer was then selectively epitaxially grown on the regrowth layer using MPCVD (process VIII). All of the superconducting samples in this study were synthesised using the same MPCVD conditions, and the thickness was controlled by changing the growth time. Finally, the metal mask was removed by acid treatment (process IX). The details of the lithography, oxygen plasma etching, and MPCVD conditions are described in the Methods section.

**Figure 2:**
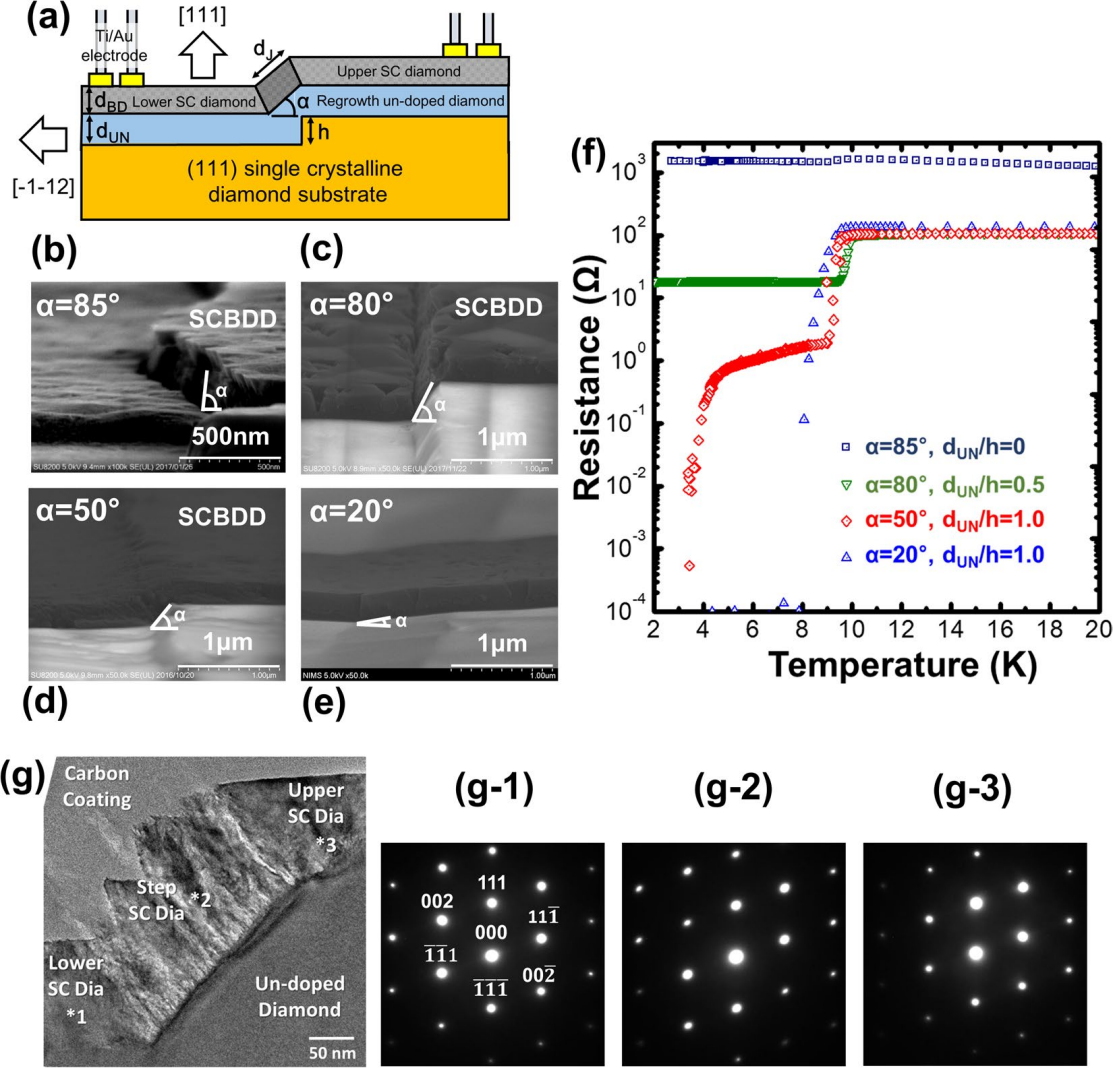
(**a**) Schematic diagram of cross-section of regrowth-induced step edge structure. (**b**–**e**) SEM images of the step edge structures with step angles α of 85°, 80°, 50°, and 20°. (**f**) Temperature dependence of the resistance of the superconducting diamond step edge structure for α of 85°, 80°, 50°, and 20°. (**g**) Cross-sectional TEM images of the fabricated regrowth-induced step edge structure. (g-1)–(g-3) are the diffraction patterns from the lower, step, and upper superconducting layers, respectively. The asterisks in (**g**) indicate the locations where the diffraction patterns were obtained.

**Figure 3:**
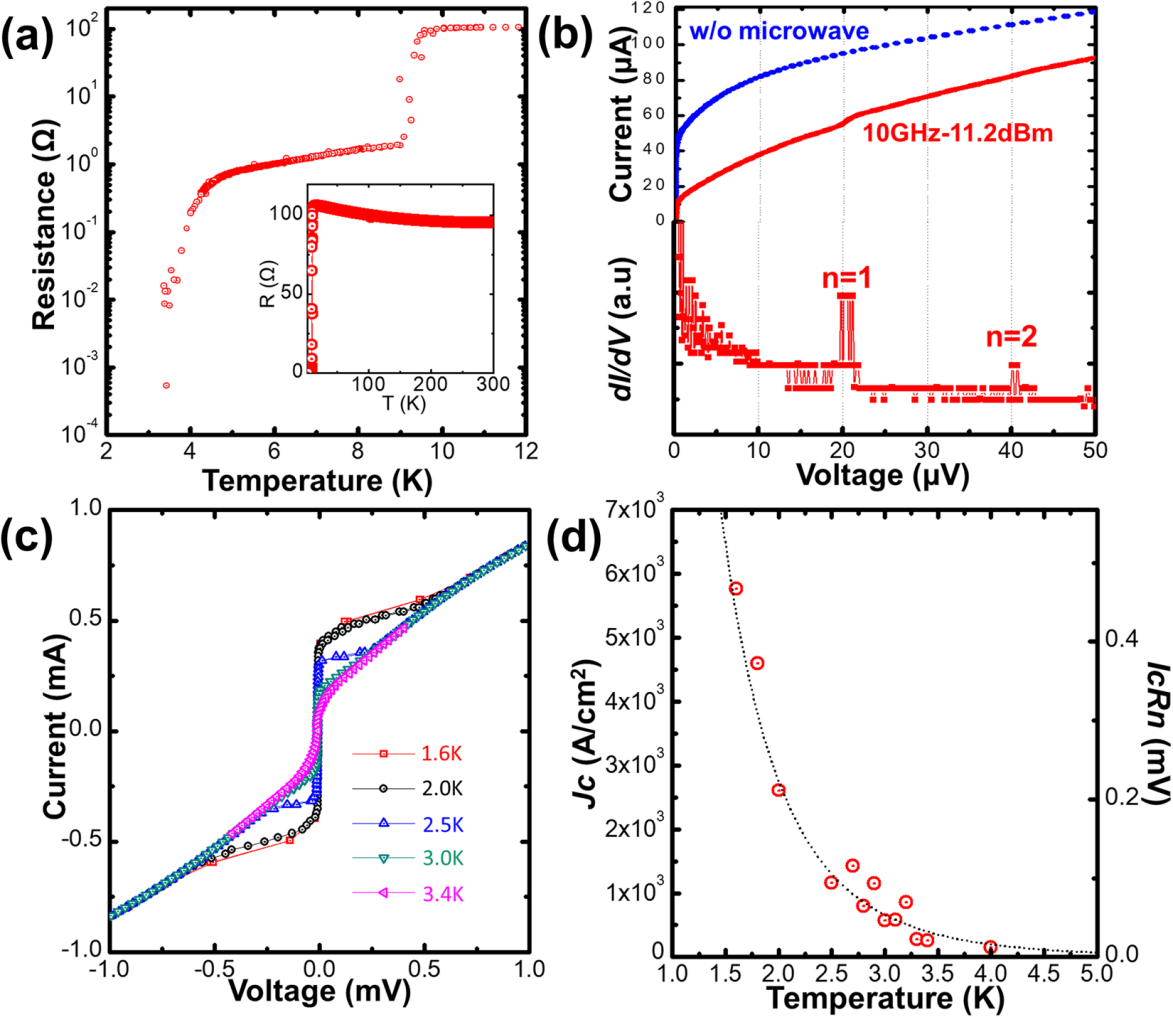
Transport properties of the diamond Josephson junction with step angle of 50°. (**a**) Temperature dependence of the resistance. The inset shows the measurements from 300 K to 2 K. (**b**) *I*–*V* characteristics at 2.8 K with and without an applied radio frequency power of −11.2 dBm at 10 GHz. (**c**) *I*–*V* characteristics from 1.6 K to 3.4 K. (**d**) Temperature dependence of the critical current density and *I*_*C*_*Rn* product.

The step angle (α), which is defined here as the angle between the surface of the substrate and the step, as shown in Fig. 2a, is a significant parameter for forming a diamond Josephson junction. We controlled the step angle by controlling the MPCVD conditions and the ratio of the thickness of the regrowth undoped layer (d_UN_) and etched step height (h). It has previously been reported that the lateral growth of (111) diamond can be achieved by using a low methane concentration (~0.05%)^35^; hence, we used similar growth conditions. Details of the MPCVD conditions for the regrowth layer are provided in the Methods section. Using lateral growth conditions, α theoretically decreases with increasing d_UN_/h. To investigate the effect of the step angle on the superconductivity, we fabricated superconducting boron-doped diamond step edge structures with step angles of 85°, 80°, 50°, and 20°. The step angles of 85°, 80°, and 50° were achieved by controlling d_UN_/h to be 0, 0.5, and 1.0, respectively. The step angle of 20° was achieved by adding CO_2_ gas during the regrowth with d_UN_/h of 1.0; the details are described in the Methods section. Here, we note that the in-plane orientation was identified as [−1, −1, 2] from pole figure measurements obtained using X-ray diffraction (Cu Kα, D8 Discover Hi-STAR, Bruker, U.S.). The structural parameters and transport properties are summarised in Table 1. Figure 2b–e presents high-resolution scanning electron microscopy (SEM) images of the fabricated step edge structures with step angles of α = 85°, 80°, 50°, and 20°. The white area in front is the surface of the regrowth layer, and the black area on the far side is the surface of the superconducting boron-doped diamond layer. An obvious crack appeared at the interface between the upper superconducting boron-doped layer and lower superconducting layer with a step angle of α = 85°. This crack was also observed for the step angle of α = 80°. However, the upper superconducting diamond film and lower superconducting film were smoothly connected for step angles of α = 50° and α = 20°. These results suggest that the crack was induced by the formation of the steep step angle. These cracks strongly affected the transport properties of the step edge structure. Figure 2f shows the temperature dependence of the resistivity of the step edge structures with step angles of α = 85°, 80°, 50°, and 20°. The step edges with step angles of α = 85° and 80° showed a one-step superconducting transition at 9.8 K and 10.0 K, respectively; however, they did not exhibit zero resistivity with residual resistance values of 1624 and 107 Ω at 4.2 K, respectively. This transition behaviour suggests that the upper and lower superconducting diamond layers simultaneously reached the superconducting state and that the step region remained in the normal conducting state. However, the step edge structure with the step angle of α = 50° exhibited a two-step superconducting transition. The first superconducting transition occurred at 9.7 K; the resistance decreased from the normal resistance value of 106 Ω at 9.7 K to 1.8 Ω at 9.2 K. Then, the resistance gradually decreased with decreasing temperature until 4.2 K; finally, the resistance dropped to the measurement limit at 3.4 K. Considering these resistance values, the upper boron-doped layer and lower boron-doped layer simultaneously reached a superconducting state at 9.2 K; however, the step region remained in a normal conducting state. The step region reached a superconducting state at 3.4 K. The step edge structure with a step angle of α = 20° exhibited a one-step superconducting transition at 9.3 K, and the resistivity dropped to zero at 7.9 K. Considering the above *R*–*T* behaviour, the following relationship between the step angle and superconducting characteristics was proposed: for a large step angle (α ≥ 80°), a crack appears between the upper and lower superconductor superconducting film, and this crack becomes a barrier; hence, the upper and lower superconducting layers are disconnected and a residual resistance appears. For a small step angle (α ≤ 20°), the upper and lower superconducting layers are completely electrically connected. They thus behave as one superconductor; hence, a Josephson junction is not formed. For a moderate step angle (α ≈ 50°), the step edge structure undergoes a two-step superconducting transition, and the Josephson junction appears to be formed at the step interface. Here, we investigated the crystallinity of the step-edge structure using cross-sectional transmission electron microscopy (TEM). Figure 2g presents a typical cross-sectional TEM image of the step-edge region with α ≈ 50°. The clear shape of the regrowth-induced step structure and the formation of a smooth upper/lower undoped diamond film is observed. The S/S′ interface coalesced homoepitaxially. This feature is beneficial for a S–S′–S Josephson junction made of diamond. Electron diffraction data (Fig. 2g-1–3) were measured at the lower, step, and upper boron-doped layer, respectively. Clear single spot diffraction patterns are observed, which indicates the high crystallinity of the boron-doped layer on the step structure. The step angle was estimated to be 49°, which corresponds to the theoretical angle of 54.7° between the [111] and [001] directions. The diffraction patterns in Fig. 2g-2 also confirm this estimation. The first *T*_*C*_(onset) of 9.7 K and second *T*_*C*_(onset) of 4.2 K also suggest the formation of a (001) plane at the step region, as previous research has revealed that the *T*_*C*_ values of (111) and (001) diamond with a boron concentration of ~1 × 10^22^ cm^−3^ are 10 K and 4 K, respectively^21^.

**Table 1:** Sample parameters, including the height of the etched step h, thickness of the undoped layer, thickness of the superconducting boron-doped layer, onset and offset first transition temperatures, onset and offset second transition temperatures, residual resistance at 2.0 K, and maximum resistance in the range of 2–300 K.

Step angle α (degrees)	h (nm)	d_UN_ (nm)	d/h	d_BD_ (nm)	1^st^ *T*_*C*_^onset^ (K)	1^st^ Tc^offset^ (K)	2^nd^ Tc^onset^ (K)	2^nd^ Tc^offset^ (K)	Residual resistance (Ω)	R(max) (Ω)
85	230	0	0	160	9.8	8.7	—	—	1527	1624
80	400	200	0.5	265	10.0	9.4	—	—	18.7	107
50	230	230	1.0	180	9.7	9.2	4.2	3.4	—	106
20	300	300	1.0	310	9.3	7.9	—	—	—	134

To verify the importance of the step angle for the formation of a Josephson junction, similar structures were fabricated in different ways. Processes I–III in Fig. 1 were similar to those of a regrowth-induced step structure, and then, an undoped layer was selectively grown by MPCVD. At the edge of the selectively grown layer, a (001) surface appeared because the [001] direction is the only low-index direction between the [111] and [-1–1 2] directions. For diamond growth, only (111) and (001) surfaces appear because of their low surface energy^36^. Thus, a boron-doped diamond layer was homoepitaxially grown over the step, as shown in Fig. S1a. Then, a Josephson junction was formed using processes VII–IX in Fig. 1. Hereinafter, this structure is referred to as a bottom-up step. The results of the *R–T* measurement are shown in Fig. S1b. A clear two-step transition similar to the regrowth-induced step edge structure was observed. The mechanism of this two-step transition is considered to be similar to that of the regrowth-induced step edge structure. The composition of the Josephson junction consisting of two superconducting (111) layers and a weak-superconducting (001) sector was examined using cross-sectional TEM, as shown in Fig. S1c. The heights of the weak superconducting layer and superconducting layers were 110 and 80 nm, respectively. The length of the weak superconducting layer was approximately 80 nm. The (001)-oriented sector corresponds to the dark area. The direction of the boundary between the (001)-oriented sector and undoped diamond, namely, the angle between the [111] and [001] directions, was 55°. The left inset (c-1) of Fig. S1c presents a diffraction pattern obtained from the (001)-oriented sector, indicating that the (001)-oriented sector is single crystalline without twins. According to the TEM observation and diffraction pattern, the entire region of the bottom-up step-edge structure Josephson junction consists of a single-crystalline layer as those of regrowth-induced step edge structures. In summary, a moderate step angle (α ≈ 50°) is essential to form a diamond Josephson junction with a step edge structure, and the (001) plane is naturally formed at the step region to form this step angle.

### Transport properties of diamond josephson junction

Figure 3 shows the transport properties of the fabricated single Josephson junction with the regrowth step angle α of 50°, which is the same sample described in the previous section. Figure 3a shows the temperature dependence of the resistance in the range of 2–12 K (inset: 2–300 K). A detailed discussion was presented in the previous section. The offset second transition temperature of 3.4 K means that the operating temperature of the Josephson junction was below 3.4 K; hence, we evaluated the Josephson properties below 3.4 K, as described below. First, we demonstrated the Shapiro step, which is a specific phenomenon of a Josephson junction, by applying low-power microwave irradiation to verify that the fabricated junction was a Josephson junction. Figure 3b shows the current and conductance (*dI/dV*) as a function of voltage for the fabricated junction at 2.8 K with and without an applied radio frequency power of −11.2 dBm at 10 GHz. The *I*–*V* curve exhibits the typical over-dumped-type behaviour without microwave irradiation, and the *I*_*C*_ was measured to be 52 μA. Then, *I*_*C*_ was decreased to 10 μA, and the periodic voltage step was observed with applied microwave irradiation. The theoretical constant step voltage only depended on the frequency of the microwave (*f*) and can be represented by Vn = (n*h*/2e) × *f*, where n is an integer, *h* is the Planck constant, and e is the elementary charge. Vn = 20.7n μV was calculated for our measurement conditions. The measured step of 20 μV was in good agreement with the theoretical value, confirming that the fabricated junction was a Josephson junction. Figure 3c shows the *I*–*V* characteristics measured at different temperatures in the range of 1.6 K–3.4 K. A clear superconducting current was observed at zero voltage, and *I*_*C*_ increased with decreasing temperature. The *I*–*V* curves reveal non-hysteretic behaviour with a high critical current, which is a great advantage for fabricating high-performance Josephson devices including SQUIDs. According to the resistively capacitively shunted junctions (RCSJ) model, the capacitance (*C*) of the junction is a significant parameter for hysteresis, and the McCumber parameter *βc* = 2π*I*_*C*_*Rn*^2^*C*/Φ_0_, where Φ_0_ of 2.07 × 10^−15^ Wb is the flux quantum, should be below 1 for a general S–N–S over-dumped-type junction. The normal-state resistance of the junction (*Rn*) was estimated to be 1.25 Ω from the slope of the *I*–*V* curve (see Fig. 3c), and *I*_*C*_ was measured to be 0.37 mA at 1.6 K; therefore, *βc*/*C* was calculated to be 1.79 × 10^−12^. Hence, the capacitance of the fabricated junction was estimated to be below 0.56 pF. This small capacitance suggests that our junction type was suitable for suppressing hysteresis. We also evaluated the temperature dependence of *I*_*C*_*Rn* and critical current density (*J*_*C*_) and the results are presented in Fig. 3d. *I*_*C*_*Rn* and *J*_*C*_ exponentially increased with decreasing temperature. Our experimental data were well fitted by the classical theory^37,38^ for long S–S′–S junctions, *I*_*C*_*Rn*(T) ∝ exp ($(\,-\,{d}_{J}/\xi ^{\prime} \sqrt{Tc})\sqrt{T}$) with ${d}_{J}/\xi ^{\prime} \sqrt{Tc}=4.5$. Here, *d*_*J*_ is the thickness of the weak superconductor, and *ξ*′ is the coherent length of the weak superconductor. *d*_*J*_ of the fabricated junction was estimated to be 300 nm using the values of h = 230 nm and α = 50°. *ξ*′ of the (001) layer with *T*_*C*_ = 3.2 K was reported to be 7.89 nm^39^; therefore, ${d}_{J}/\xi ^{\prime} \sqrt{Tc}$ was calculated to be approximately 8.9. This value is consistent with our fitting parameter, confirming that the fabricated junction is an S–S′–S-type junction. The highest *I*_*C*_*Rn* and *J*_*C*_ were 0.47 mV and 5800 A/cm^2^ at 1.6 K, respectively. These values are more than ten-times higher than those previously reported^33,34^, which indicates that the step edge S–S′–S structure is better than the vertical S–N–S stack structure for single-crystalline diamond. We also investigated the transport properties of the fabricated Josephson junction with bottom-up step edge structure, and the results are summarised in Fig. S2. For the *I*–*V* characteristics at 2.5 K (Fig. S2a), a non-hysteretic curve was observed, and the temperature dependence of *J*_*C*_ showed exponential behaviour like the regrowth-induced step edge structure.

### Diamond SQUID operation

**Figure 4:**
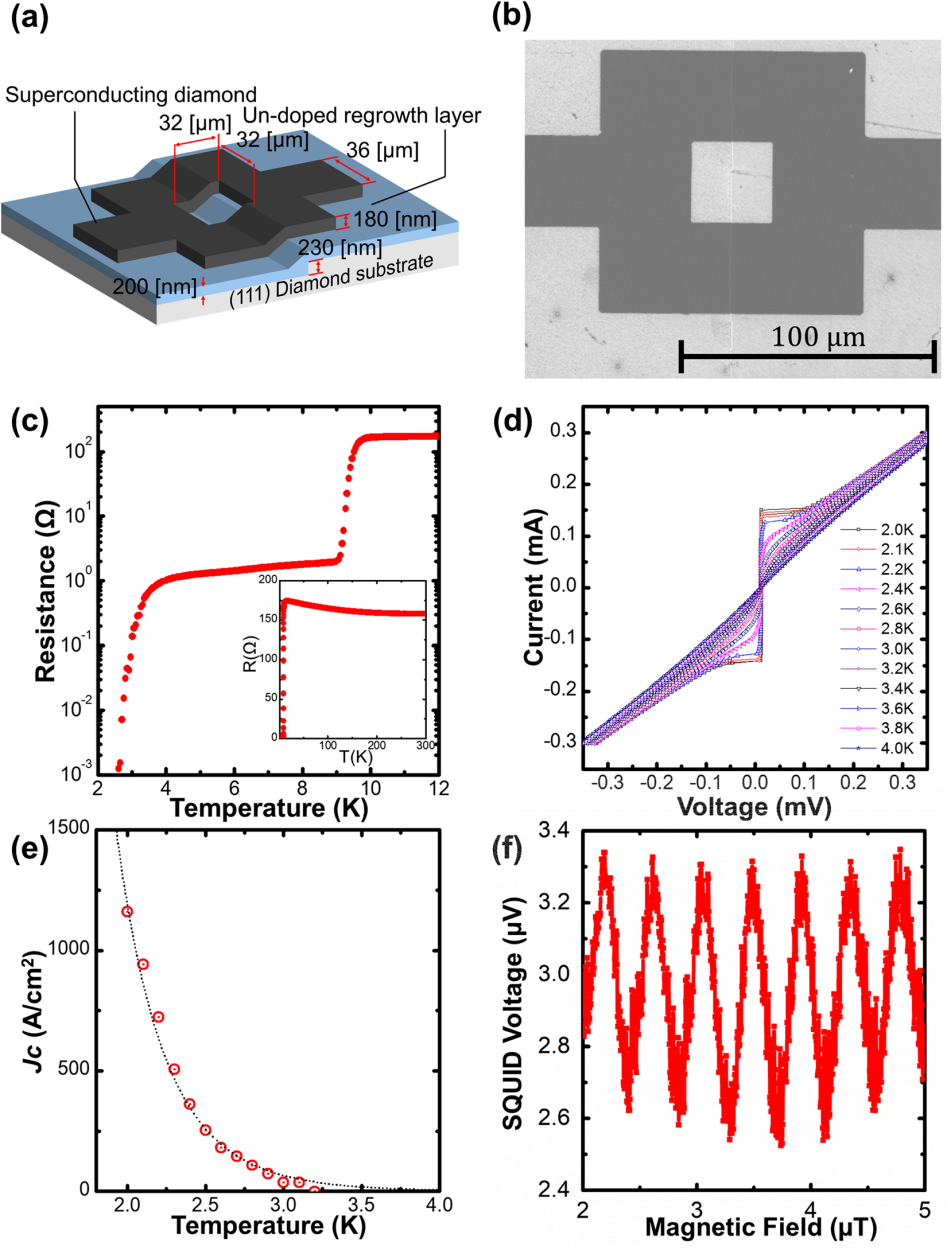
(**a**) Schematic diagram of the fabricated diamond SQUID. (**b**) Overview SEM image of fabricated SQUID. (**c**) Temperature dependence of the resistance. The inset shows the measurements from 300 K to 2 K. (**d**) *I*–*V* characteristics from 2.0 K to 4.0 K. (**e**) Temperature dependence of the critical current density. (**f**) Flux–voltage (*Φ* − *V*) characteristics of the fabricated SQUID at 2.6 K.

Finally, we investigated the transport properties of the fabricated single-crystalline diamond dc-SQUID. The device structure and structural parameters are shown in Fig. 4a, and overview SEM images of the fabricated SQUID are presented in Fig. 4b. The structural parameters were the same as those for the single Josephson junction sample: h = 230 nm, d_UN_ = 230 nm, d_BD_ = 180 nm, and α = 50°. The width of the superconducting diamond film was constant at 36 μm, and the SQUID loop area was 32 × 32 µm^2^; thus, the effective SQUID loop area in consideration of the Meissner effect was 68 × 68 µm^2^. The formation of a uniform straight step region was observed in a vertical-view SEM image. Figure 4c presents the *R*–*T* measurements of the fabricated SQUID in the range of 2–12 K (inset: 2–300 K). A clear two-step transition was observed, which was similar to the result for the Josephson junction sample in Fig. 3a. The onset/offset first transition temperatures were 10 K and 9.0 K, respectively, and the onset/offset second transition temperatures were 4.0 K and 2.8 K, respectively. The *I*–*V* characteristics of the fabricated junction at 2.0 K–4.0 K are shown in Fig. 4d. The *I*–*V* curves exhibited non-hysteresis behaviour in the range of 2.2 K–4.0 K; however, the *I*–*V* curves at 2.0 K and 2.1 K showed small hysteresis (see the Supplementary Fig. S3). This thermal hysteresis appeared to be induced by the increase of *I*_*C*_ with decreasing temperature. The temperature dependences of *J*_*C*_ are shown in Fig. 4e. *J*_*C*_ increased exponentially with decreasing temperature, and the experimental data were well fitted by *J*_*C*_(T) ∝ exp ((−*d*_*J*_/ξ′$\sqrt{Tc}$)$\sqrt{T}$) with *d*_*J*_/*ξ*′$\sqrt{Tc}$ = 9.1. *J*_*C*_ of the SQUID was lower than that of the Josephson junction sample. The main reason for this finding is that the *T*_*C*_ of the SQUID step region (2.8 K) was lower than that of the Josephson junction (3.4 K). SQUID operation was demonstrated by the flux–voltage (*Φ* *−* *V*) measurement at 2.6 K, as shown in Fig. 4f. The voltage periodically oscillated as a function of the magnetic field. The measured oscillation interval was 0.43 μT, which is in good agreement with the theoretical value of 0.45 μT calculated from Φ_0_/A_eff_; here, Φ_0_ and A_eff_ are the flux quantum and effective SQUID loop area of 68 × 68 μm^2^. The average amplitude of the voltage modulation (peak-to-peak voltage *V*_p–p_) was approximately 0.8 μV. This result confirms that the structure we fabricated operated as a SQUID.

## Conclusions

We developed a fabrication process for a single-crystalline diamond Josephson junction with a regrowth-induced step edge structure. This structure is suitable for forming an S–S′–S-type non-hysteretic Josephson junction with a high critical current density using single-crystalline diamond. We also demonstrated the first operation of a single-crystalline diamond SQUID, which resulted in improvement of the operating temperature. To further improve the operating temperature, optimisation of the step angle and height are necessary. It is possible to operate diamond SQUIDs at 10 K if a step structure consisting of only (111) planes can be realised. Although further improvements are needed, we believe that our results are the first step towards the operation of diamond SQUIDs under a simple cooling system without the need for ^3^He gas.

## Methods

### Sample preparation

In this study, commercially available (111)-oriented single-crystalline diamond synthesised using a high-pressure and high-temperature (HPHT) method was used for the substrate. The regrowth undoped diamond layer (process VI in Fig. 1) was synthesised using two different ASTeX MPCVD apparatuses. For the samples with step angles of 85°, 80°, and 50°, the methane concentration [CH_4_] was 0.05% diluted with hydrogen; the total gas flow was 400 sccm; and the gas pressure and substrate temperature during growth were 35 Torr and 620 °C, respectively. The growth rate for the above conditions was estimated to be 41.7 nm/h using secondary ion mass spectroscopy. For the sample with a step angle of 20°, the methane concentration [CH4] and CO_2_ concentration [CO_2_] were 0.75% diluted with hydrogen; the total gas flow was 400 sccm; and the gas pressure and substrate temperature during growth were 35 Torr and 600 °C, respectively. The growth rate for the above conditions was estimated to be 150 nm/h using secondary ion mass spectroscopy. The superconducting boron-doped diamond layer (process VIII in Fig. 1) was synthesised using a lab-made quartz-tube-type MPCVD apparatus. Tri-methyl-boron [B(CH_3_)_3_] was used as the dopant gas; [TMB]/[CH_4_] was 9000 ppm, [CH_4_] was 5%, the total gas flow was 100 sccm, and the gas pressure and substrate temperature during growth were 110 Torr and 760 °C, respectively. The growth rate for the above conditions was estimated to be 770 nm/h using a stylus-based profilometer. The patterning of the metal mask for O_2_ plasma etching and selective MPCVD growth of the Josephson junction and SQUID was performed using laser maskless lithography (DL-1000, Nanosystem Solutions, Japan) (process I VII in Fig. 1) and EB evaporation (process II VII in Fig. 1). LOR5A and AZ5214E were used as the photoresists. Before laser lithography, the sample surface was cleaned using boiled acid treatment (1:3 mixture of HNO_3_ and H_2_SO_4_ at 200 °C for 30 min) followed by rinsing with deionised water and terminated with oxygen by exposure to ultraviolet (UV) light in an oxygen environment for 3 h at room temperature. O_2_ inductive coupled plasma reactive ion etching (ICP-RIE) was performed to form a vertical step (process V in Fig. 1). The O_2_ gas flow was 45 sccm; the gas pressure was 1.4 Pa; and the power of the antenna and bias were 700 and 100 W, respectively. The etching rate for the above conditions was estimated to be 220 nm/min using a stylus-based profilometer. After fabricating the Josephson junction and SQUID, four titanium and gold (Ti/Au: 30/100 nm thickness) contact pads were deposited by EB evaporation as shown in Fig. 2e, and the sample was annealed at 500 °C in a vacuum greater than 1 × 10^−5^ Pa to obtain good adhesion between the diamond surface and titanium.

### Measurement setup

The surface morphologies of the step structure were examined using high-resolution field-emission scanning electron microscopy (FE-SEM) (S4800/SU-8240, HITACHI High-Technologies Corp., Japan). A high-resolution cross-sectional transmission electron microscopy (HR-TEM) image was obtained using field-emission transmission electron microscopy (FE-TEM; JEM 2100F, JEOL, Japan) with an accelerating voltage of 200 kV. The cross-section for TEM observation was prepared using a focused ion beam system (JIB-4000, JEOL, Japan). The transport properties were characterised using a physical property measurement system (PPMS, Quantum Design, U.S.) and magnetic property measurement system (MPMS, Quantum Design, U.S.). The electrical resistances were measured using the four-probe method. In this work, the critical current density (*I*_*C*_) was defined as the current at which the measured voltage reached 1 μV. The critical current density (*J*_*C*_) was given by *J*_*C*_ = *I*_*C*_/*S*, where *S* is the cross-sectional area of the superconducting diamond film. In the SQUID operation, the SQUID was biased at constant DC current above the critical current using a function generator, and a magnetic field was applied in a perpendicular configuration from a coil on the back side of the sample holder.

## Supplementary information

Supplementary Information
