# Supplementary material for: Single-crystalline boron-doped diamond superconducting quantum interference devices with regrowth-induced step edge structure
Source: Sci Rep. 2019 Oct 23;9:15214. doi: 10.1038/s41598-019-51596-w (PMC6811626; doi:10.1038/s41598-019-51596-w)
Supplement: Supplementary file 1 — Supplementary Information [file 41598_2019_51596_MOESM1_ESM.docx]

**Supplementary Information**

**Single-crystalline boron-doped diamond superconducting quantum interference devices with regrowth-induced step edge structure**

**Taisuke Kageura^1*^, Masakuni Hideko^1^, Ikuto Tsuyuzaki^1^, Aoi Morishita^1^, Akihiro Kawano^1^, Yosuke Sasama^2^, Takahide Yamaguchi^2^, Yoshihiko Takano^2^, Minoru Tachiki^2^, Shuuichi Ooi^2^, Kazuto Hirata^2^, Shunichi Arisawa^2^ and Hiroshi Kawarada^1,3*^**

^1^ Faculty of Science & Engineering, Waseda University, 3-4-1, Okubo, Shinjuku-ku, Tokyo, 169-8555, Japan

^2^ National Institute for Materials Science, 1-2-1, Sengen, Tsukuba, Ibaraki, 305-0047, Japan

^3^ The Kagami Memorial Laboratory for Materials Science and Technology, Waseda University, 2-8-26 Nishiwaseda, Shinjuku-ku, Tokyo 169-0051, Japan

*Tel: +81-3-5286-3391; E-mail: tai0723@fuji.waseda.jp, kawarada@waseda.

**
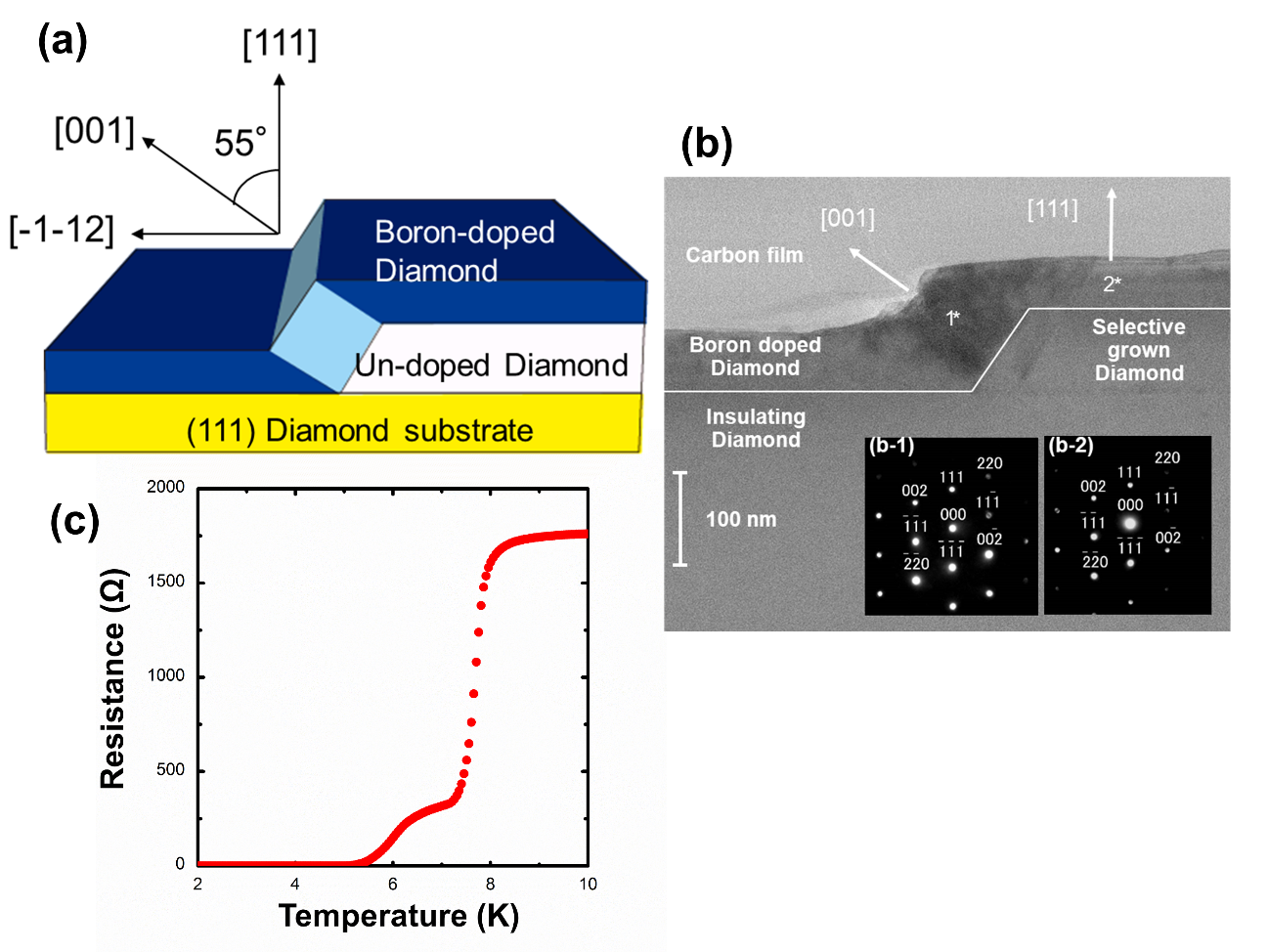
Figure S1.** (a) Schematic diagram of cross-section of bottom-up step edge structure. (b) Cross-sectional TEM images of fabricated bottom-up step edge structure. Insets (b-1) and (b-2) show the diffraction patterns from the step and upper superconducting layers, respectively. The asterisks in (b) indicate the locations where the diffraction patterns were obtained. (c) Temperature dependence of the resistance of the bottom-up step edge structure.

**
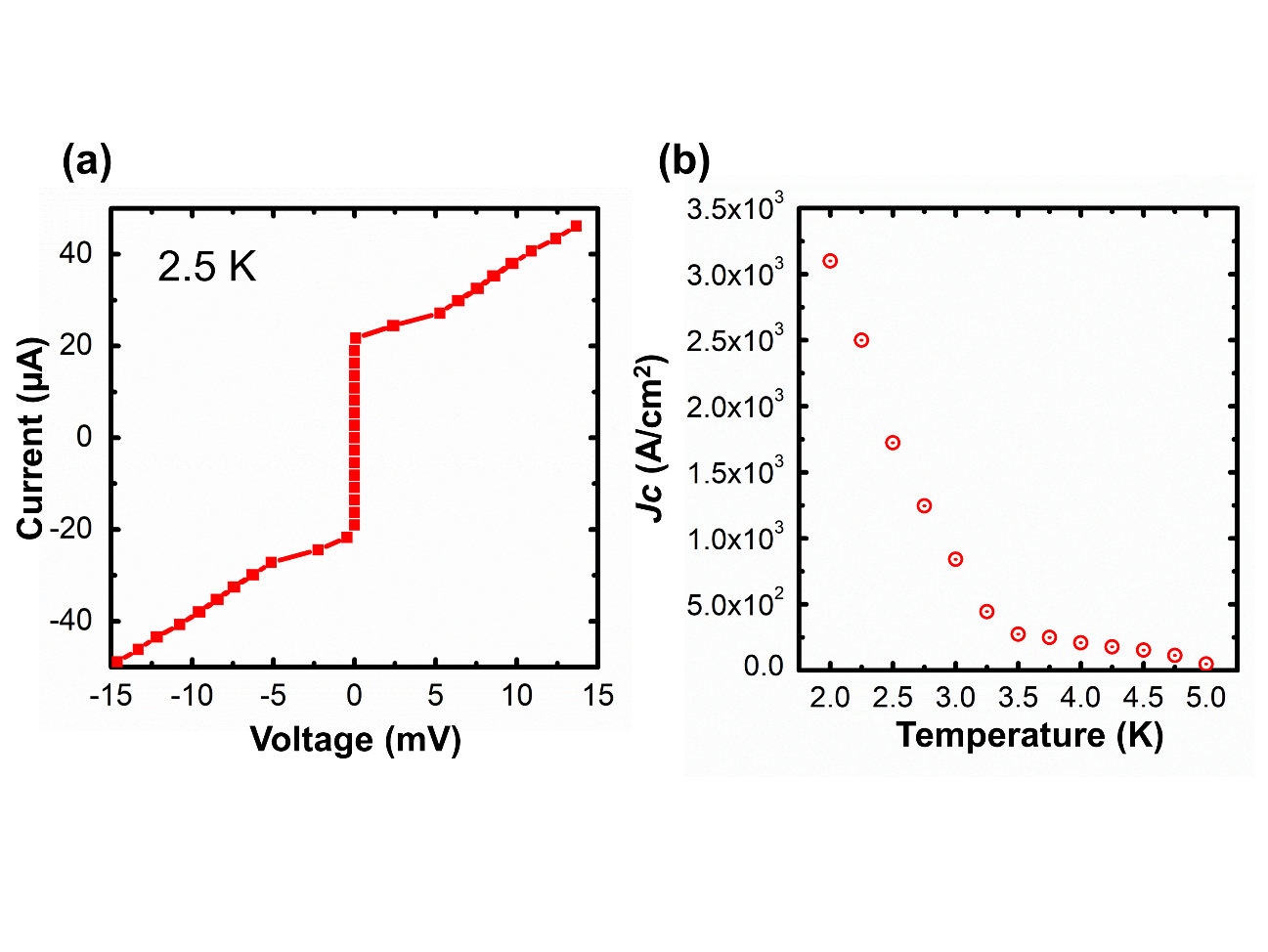
**

**Figure S2.** (a) *I–V* characteristics of the bottom-up step edge structure at 2.5 K. (b) Temperature dependence of the critical current density of the bottom-up step edge structure.

**
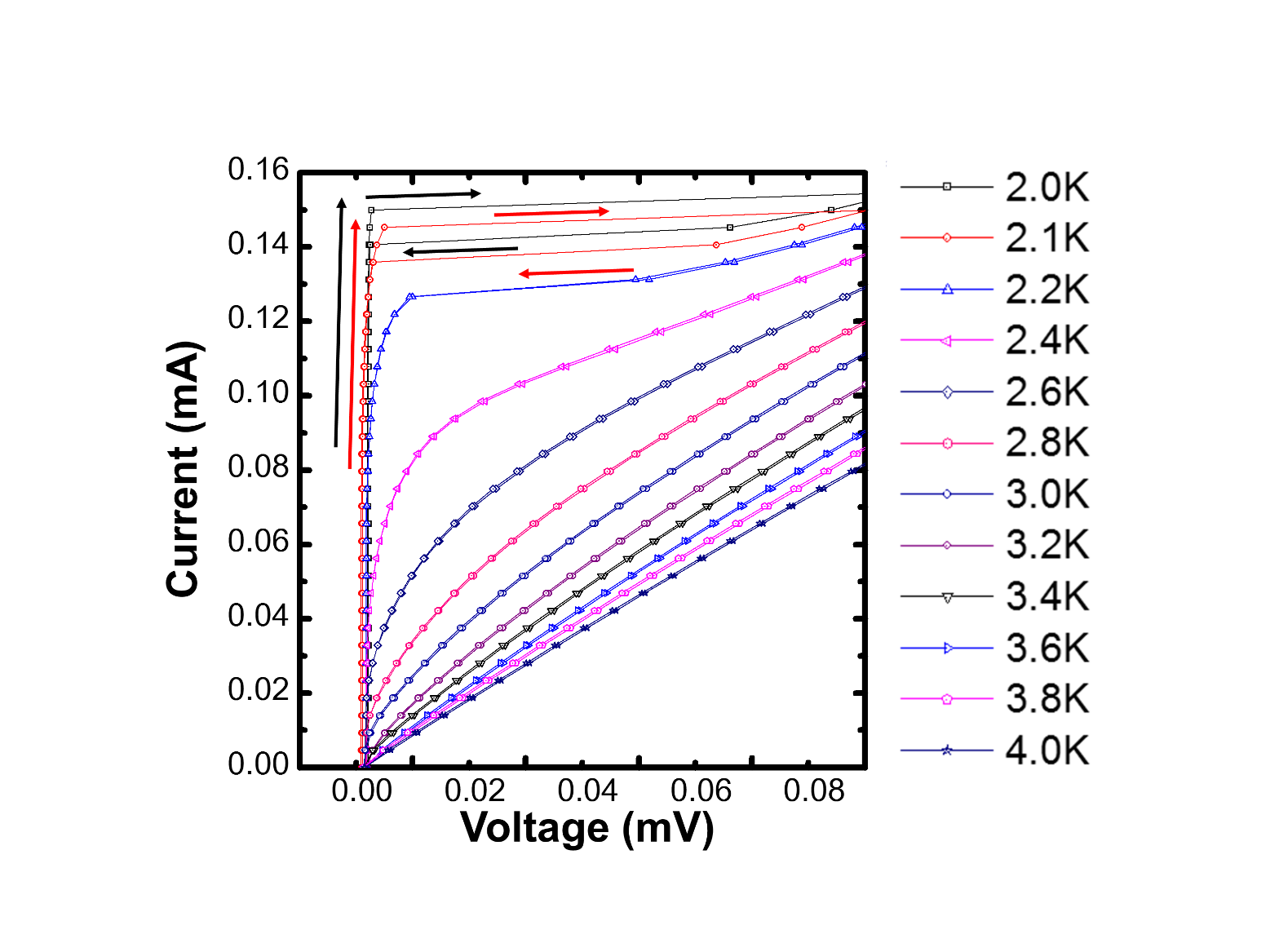
**

**Figure S3.** Enlarged *I*–*V* characteristics of Fig. 4d. The arrows indicate the measurement order. The *I–V* curves at 2.0 K and 2.1 K show small hysteresis, whereas no hysteresis is observed in the *I–V* curves at 2.2 K to 4.0 K.
